# Prevalence and risk factors for esophageal squamous cell cancer and precursor lesions in Anyang, China: a population-based endoscopic survey

**DOI:** 10.1038/sj.bjc.6605843

**Published:** 2010-08-10

**Authors:** Z He, Y Zhao, C Guo, Y Liu, M Sun, F Liu, X Wang, F Guo, K Chen, L Gao, T Ning, Y Pan, Y Li, S Zhang, C Lu, Z Wang, H Cai, Y Ke

**Affiliations:** 1 Key Laboratory of Carcinogenesis and Translational Research (Ministry of Education), Laboratory of Genetics, Peking University School of Oncology, Beijing Cancer Hospital & Institute, 52 Fucheng Rd. Hai Dian District, 100142 Beijing, People’s Republic of China; 2 Anyang Cancer Hospital, Henan Province, People’s Republic of China

**Keywords:** prevalence, risk factor, esophageal cancer, precursor lesion, China

## Abstract

**Background:**

The etiology of esophageal squamous cell cancer (ESCC) in high prevalence regions of China remains unclear.

**Methods:**

Endoscopic biopsies were conducted among 7381 inhabitants aged from 25 to 65 of Anyang, China.

**Results:**

In this study, 2.57, 0.20 and 0.16% of the participants had mild, moderate and severe squamous dysplasia, respectively; 0.19 and 0.08% showed squamous carcinoma *in situ* and invasive ESCC. Using deep well (depth >100 meters) as water source (odds ratio=0.72, 95% confidence interval: 0.54–0.96) was negatively associated with ESCC and its precursors, whereas tobacco and alcohol use were not significantly associated with ESCC.

**Conclusions:**

Water source and other factors in this region need further evaluation by longitudinal studies.

Esophageal cancer is the sixth most common cancer among men and ninth most common among women (WHO & IARC, 2008). Tobacco and alcohol use are well recognized as the main risk factors for esophageal cancer in western countries (Castellsague *et al*, 2000; Adami *et al*, 2002).

In Anyang, a region in north central China, mortality from esophageal squamous cell cancer (ESCC) is among the highest in the world (Ke, 2002; WHO & IARC, 2008) being 10-fold greater than the nationwide rate for China and 100-fold greater than the rate among Caucasian Americans (Blot and Li, 1985). Beginning in the 1980s, a series of epidemiology studies to identify the risk factors in this area were carried out in Linzhou (formerly Linxian or Lin County), Anyang. These indicated that cigarette smoking, tooth loss and lack of certain nutrients and minerals (e.g. selenium) were associated with higher risk of ESCC (Mark *et al*, 2000; Abnet *et al*, 2001; Tran *et al*, 2005; Wei *et al*, 2005; Qiao *et al*, 2009). However, the discrimination model for ESCC that was constructed on the basis of these determinants performed suboptimally, with sensitivity and specificity values of 57 and 54%, respectively (Wei *et al*, 2005), strongly suggesting that other pathogenic factors had not been effectively evaluated.

In 2007, we initiated a population-based esophageal cancer cohort study in Anyang, China using endoscopic biopsy and a questionnaire investigation to determine its age specific prevalence and that of its precursor lesions in this region, and investigate the possible risk factors for ESCC. The results would also serve as baseline data for our subsequent longitudinal investigation.

## Materials and methods

Anyang, an agricultural region in the northern part of Henan Province, PR China, consisting of five counties, Linzhou, Hua County, Anyang County, Tangyin County and Neihuang County, with a population of 5.37 million; its gross domestic product (GDP) per capita is about US $1760. In 2007–09, we conducted an endoscopic survey for esophageal cancer and precursor lesions in nine villages of rural Anyang, which were selected on the basis of the population size and location. Five were in Hua County, two in Linzhou, one in Anyang County and one in Tangyin County. Criteria for participant eligibility were as follows: (1) permanent residency in one of these villages; (2) age 25 to 65; (3) no previous cardiocerebral vascular diagnoses or psychological disorders; (4) voluntary participation in this study and agreement to complete all phases of the examination, including endoscopy.

Endoscopy was performed with Lugol’s iodine staining. The entire esophagus and stomach were visually examined, and biopsies were taken from all focal lesions identified. A standard site in the mid-esophagus (25 cm distal to the incisors in the 6 o’clock position) was sampled if no abnormalities were found. Biopsies were fixed in 10% formaldehyde, embedded in paraffin, sectioned at 5 mm, and stained with hematoxylin and eosin. Biopsy slides were read independently by two pathologists at Anyang Cancer Hospital, without knowledge of the subjects’ history or endoscopic findings. Diagnoses included normal, esophagitis, basal cell hyperplasia (BCH), squamous dysplasia (mild, moderate and severe), squamous carcinoma *in situ* (CIS), invasive squamous cell carcinoma and adenocarcinoma based on commonly accepted criteria (Dawsey *et al*, 1994; Wei *et al*, 2005).

A questionnaire was completed covering socio-demographic characteristics, pesticide exposure, living environment, alcohol and tobacco use and dietary habits. Interviews were carried out one-on-one in a room situated so as to ensure privacy.

### Statistical analysis

Crude detection rates of all histological diagnoses were standardized by the age structure of the World Health Organization world standard population of 1985 (Ahmad *et al*, 2000). Differences in detection rate among groups were evaluated using the *χ*^2^-test and Fisher’s exact test. Univariate and multivariate unconditional logistic regression was employed to identify risk factors for ESCC and its precursor lesions. All subjects were categorized either as ‘case group’ (all grades of dysplasia, CIS and ESCC) or ‘control group’ (normal epithelium, esophagitis and BCH) for logistic regression analysis. Two cases of adenocarcinoma were excluded from this analysis. All statistical analyses were conducted using SPSS for Windows version 11.5. All *P*-values were two-sided and *P*-values less than 0.05 were considered statistically significant.

### Ethical review

An individual informed consent was signed by all participants. This study was approved by the Institutional Review Board of the School of Oncology, Peking University, China.

## Results

**Figure 1:**
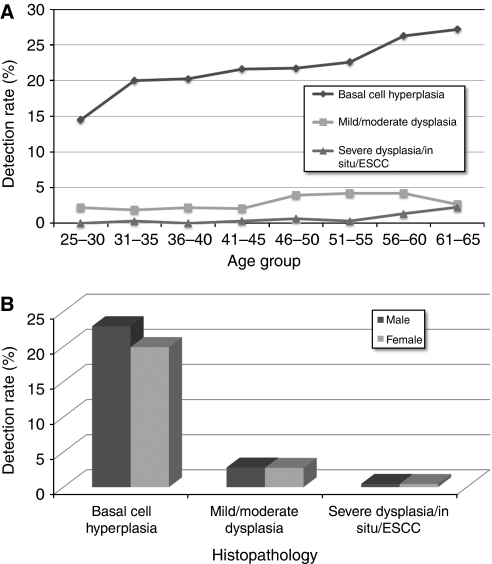
(**A**) Detection rates of esophageal cancer and precursor lesions by age. (**B**) Detection rates of esophageal cancer and precursor lesions by gender.

A total of 10439 residents were eligible and 7643 (73.22%) completed all required blood testing, endoscopy and biopsy and the questionnaire. Of these individuals, 7381 (96.57%) had at least one biopsy technically adequate for pathology diagnosis. As a recent study in this region demonstrated that esophagitis was not associated with risk of esophageal cancer (Wang *et al*, 2005), ‘normal’ and ‘esophagitis’ diagnoses were combined into one category. For all 7381 subjects, 5568 (75.44%) were diagnosed as normal or esophagitis; 1574 (21.33%) had BCH; 190 (2.57%) showed mild squamous dysplasia; 15 (0.20%) had moderate squamous dysplasia; 12 (0.16%) had severe squamous dysplasia; 14 (0.19%) had CIS; 6 (0.08%) had squamous cell carcinoma; and 2 (0.03%) had adenocarcinoma (Table 1). Figure 1a shows the age specific detection rates of lesions ranging from BCH to ESCC. Detection rates of BCH increased monotonically with age in all age groups. Detection rates of mild/moderate dysplasia were higher than that of severe dysplasia/CIS/ESCC in all age groups. However, mild/moderate dysplasia began to decrease in the 51–55 year group, whereas detection rates of severe dysplasia/CIS/ESCC showed a concomitant significantly increasing trend and these two curves almost intersected in the 61–65 year group. More BCH was found in males (22.92%) than in females (19.94%, *P*=0.002) and there were no other significant gender differences in detection rates of esophageal cancer or its precursor lesions (Table 1, Figure 1b).

Potential ESCC risk factors, including tobacco and alcohol use were evaluated using unconditional logistic regression analysis (Table 2). Univariate logistic regression showed that tooth loss (*P*_*trend*_=0.017) was associated with increased risk of ESCC and its precursors, whereas use of water pumped from a deep well (over 100 m depth) (odds ratio=0.72, 95% confidence interval=0.54–0.95, *P*=0.022) showed a negative association with ESCC and its precursor lesions. After adjusting for age and sex, use of deep well water remained as having a negative association with ESCC and precursor lesions (odds ratio=0.72, 95% confidence interval=0.54–0.96; *P*=0.023). Cigarette smoking, alcohol intake, pesticide exposure and hot food intake were not found to correlate with ESCC and precursor lesions.

## Discussion

The Anyang region of China is a high-risk region for ESCC, and a series of studies on ESCC have been carried out there. To obtain the greatest number of patients with ESCC or precursor lesions as possible, participants chosen for almost all these studies were of generally greater age (Li *et al*, 1989; Dawsey *et al*, 1994; Tran *et al*, 2005; Wang *et al*, 2005; Wei *et al*, 2005). These studies, therefore, typically reported much higher rates of cancer and precursor lesions. For example, rates of mild, moderate and severe esophageal dysplasia of 8.6, 7.8 and 2.6%, respectively, were recently reported in Cixian, an adjacent high-risk region (Lu *et al*, 2004). In our population-based screening study we evaluated data over a broader age range (25–65 years), and included individuals from entirety rural area around Anyang. This age difference is important given the positive association with age of ESCC and precancerous lesions. We consider the prevalence of ESCC and its precursors in younger age groups is important for etiologic studies in this high-risk region, as the cumulative effects of pathogenic factors (e.g. water source) may be evaluated more efficiently through analysis with full consideration and adjustment for age.

Water source is not a common risk factor for ESCC, especially in developed countries. Until about 20 years ago, the main sources of drinking water in rural area of Anyang were rivers or shallow wells. However, since the 1980s, the local government has committed significant resources to an extensive water improvement project, and now-a-days deep groundwater has become the main water source for most of the inhabitants of rural Anyang. A previous ecologic study reported that the incidence and mortality from esophageal cancer in Linzhou showed a decreasing trend in villages included in this water improvement project as compared with those without water improvement (Han *et al*, 2007). The conclusions of ecological studies cannot be directly linked to the etiology of disease, but in the current population-based study, we found that use of deep well water had an independent negative association with ESCC and precancerous lesions. This is the first population-based study to suggest an association between water source and ESCC. Although further confirmation and studies of the mechanism will be necessary, these findings could be taken as evidence for the promotion of etiologic research and ESCC control in economically underdeveloped regions where surface or shallow ground water are still used as a main water source.

Although tobacco and alcohol are well-established risk factors for ESCC in western countries (Adami *et al*, 2002; IARC, 2004; Freedman *et al*, 2007; Pandeya *et al*, 2008) this is neither the case in Asian high-risk regions including Anyang (Cook-Mozaffari *et al*, 1979; Yu *et al*, 1993; Guo *et al*, 1994) nor in our study. Tooth loss has been reported to be associated with an increased risk of esophageal cancer (Wang *et al*, 1992; Abnet *et al*, 2001, 2005) Changes in chewing may expose the esophagus to more frequent injury and may also result in increased risk of oral bacterial infection and production of carcinogens, such as nitrosamines (Abnet *et al*, 2001; Guha *et al*, 2007). In our analysis, tooth loss was positively associated with ESCC in the univariate analysis, but not in the multivariate analysis. Subsequent multivariate analysis stratified by age showed a clear association with tooth loss in the group less than 50 years old, but not in older subjects (data not shown). We believe that both tooth loss itself and lack of retrospective information about tooth loss history are likely to have contributed to these age differences, and to have biased the relationship toward the null hypothesis in the older age groups. We consider that tooth loss in early life is likely to be associated with ESCC in Anyang.

In summary, 7381 individuals of rural Anyang participated in this cross-sectional endoscopic survey. Detection rates of mild, moderate and severe squamous dysplasia, CIS and ESCC were 2.57, 0.20, 0.16, 0.19 and 0.08%. Use of water pumped from deep wells was negatively associated with ESCC and precursor lesions, whereas tobacco and alcohol use had no significant relationship with ESCC. Longitudinal studies are needed of other risk factors that may contribute to ESCC in this high-risk region.

## Figures and Tables

**Table 1:** Detection rates of esophageal cancer and precursor lesions by gender

	**Total (*N*=7381)**			**Male (*N*=3425)**		**Female (*N*=3956)**		
**Histopathology**	***n***	**%**	**% (Adjusted)^a^**	***n***	**%**	***n***	**%**	***P* b**
Normal or esophagitis	5568	75.44	76.19	2528	73.81	3040	76.85	0.003
Basal cell hyperplasia	1574	21.33	20.67	785	22.92	789	19.94	0.002
Squamous dysplasia								
Mild	190	2.57	2.53	89	2.60	101	2.55	0.902
Moderate	15	0.20	0.17	7	0.20	8	0.20	0.984
Severe	12	0.16	0.15	3	0.09	9	0.23	0.137
Squamous carcinoma *in situ*	14	0.19	0.19	7	0.20	7	0.18	0.787
Squamous cell carcinoma	6	0.08	0.08	5	0.15	1	0.03	0.160
Squamous cell carcinoma and precursor lesions	237	3.21	3.12	111	3.24	126	3.19	0.892
Adenocarcinoma	2	0.03	0.02	1	0.03	1	0.03	1.000

a Detection rates were adjusted by standard age structure of the world population (WHO, 1985).

b *P*-values were derived by *χ*^2^-test or Fisher's exact test.

**Table 2:** Univariate and multivariate unconditional logistic analysis of risk factors associated with esophageal squamous cell carcinoma and precursor lesions

	**Subjects with ESCC and**	**Subjects without ESCC and**	**Unadjusted**	**95%CI**				**Adjusted**	**95%CI**			
**Variables**	**precursor lesions *n* (%)**	**precursor lesions *n* (%)**	**odds ratio**	**Lower**	**Upper**	***P***	***P-*trend^a^**	**odds ratio^b^**	**Lower**	**Upper**	***P***	***P-*trend^a^**
*Smoking history*												
Never	161 (70.61)	4934 (71.18)	1.00	—	—	Ref.		1.00	—	—	Ref.	
Current	60 (26.32)	1713 (24.71)	1.07	0.79	1.45	0.645		1.00	0.67	1.50	0.998	
Former	7 (3.07)	285 (4.11)	0.75	0.35	1.62	0.467		0.62	0.27	1.40	0.246	
												
*Drinking history*												
Never	204 (89.47)	5995 (86.52)	1.00	—	—	Ref.		1.00	—	—	Ref.	
Current	23 (10.09)	902 (13.02)	0.75	0.48	1.16	0.195		0.71	0.45	1.14	0.158	
Former	1 (0.44)	32 (0.46)	0.92	0.12	6.75	0.933		0.83	0.11	6.16	0.854	
												
*Tooth loss*												
⩽4	23 (48.94)	1677 (70.43)	1.00	—	—	Ref.	0.018	1.00	—	—	Ref.	0.809
5–9	14 (29.79)	391 (16.42)	2.61	1.33	5.12	0.005		1.86	0.90	3.87	0.095	
10–14	5 (10.64)	129 (5.42)	2.83	1.06	7.56	0.038		1.59	0.54	4.68	0.397	
⩾15	5 (10.64)	184 (7.73)	1.98	0.74	5.27	0.171		0.97	0.32	2.93	0.963	
												
*Pesticide exposure*												
No	38 (16.67)	1187 (17.12)	1.00	—	—	Ref.		1.00	—	—	Ref.	
Yes	190 (83.33)	5745 (82.88)	1.03	0.73	1.47	0.857		1.00	0.70	1.44	0.991	
												
*Preferred food temperature*												
Cold	24 (10.53)	843 (12.17)	1.00	—	—	Ref.	0.102	1.00	—	—	Ref.	0.135
Moderate	115 (50.44)	3758 (54.24)	1.07	0.69	1.68	0.751		0.98	0.62	1.54	0.929	
Hot	89 (39.04)	2328 (33.60)	1.34	0.85	2.12	0.207		1.27	0.80	2.01	0.314	
*Water source*												
Shallow well	151 (66.81)	4067 (59.17)	1.00	—	—	Ref.		1.00	—	—	Ref.	
Deep motor-pumped well	75 (33.19)	2806 (40.83)	0.72	0.54	0.95	0.022		0.72	0.54	0.96	0.023	

Abbreviation: CI=confidence interval.

a To evaluate trends in odds, ordinal variables were modeled as single, continuous, independent variables.

b Odds ratios were adjusted for age (5-year groups) and sex.
